# Intravitreal administration of bevacizumab: pros and cons

**DOI:** 10.1186/s40199-015-0110-0

**Published:** 2015-04-21

**Authors:** Simin Dashti-Khavidaki, Mohammad Abdollahi

**Affiliations:** Faculty of Pharmacy, Tehran University of Medical Sciences, Tehran, Iran; Faculty of Pharmacy and Pharmaceutical Sciences Research Center, Tehran University of Medical Sciences, Tehran, 1417614411 Iran

## Bevacizumab uses

Bevacizumab (Avastin®, Genethech, Inc.) is a humanized monoclonal antibody that inhibits vascular endothelial growth factor (VEGF). It was first approved by the US food and drug administration for metastatic colorectal cancer and subsequently for cervical/ovarian/fallopian tube cancers, glioblastoma, non-small cell lung cancer, and metastaic renal cell carcinoma [1]. Recently its off-label use for age-related macular degeneration (AMD) has received extensive attention. Single dose vial of intravitreal ranibizumab (IVR), another anti-VEGF antibody, has been licensed for treatment of wet AMD and diabetic macular edema [1,2]. Due to lower cost and reported equall efficacy and safety data in large clinicat trials such as The Comparison of Age-related Macular Degeneration Treatment Trial (CATT), The Inhibitors of VEGF in Age-related Choroidal Neovascularization (IVAN) and GEFAL studies, intravitreal bevacizumab (IVB) has replaced the intravitreal administration of the approved drug ranibizumab [2-7]. A recent review on IVB administration for management of AMD and pathological myopathy concluded that despite promising results, short-term patients’ follow-up in the available studies, lack of well-designed extensive clinical trials, and insufficient data on safety of IVB make its use cautiously in these ocular diseases [8]. IVB administration has been widely used in other different proliferative retinopathies including retinopathy of prematurity [9,10] and diabetic retinopathy [9,11] as well. Angiogenesis with key regulator VEGF is the main pathology in these retinal and choroidal diseases [9]. A review on IVB administration for treatment of different aforementioned retinal and choroidal diseases showed promising reults [12].

## IVB complications

Two categories of complications have been reported for IVB: post-injection and drug class-related complications. Post-injection complications that are not influenced by the underlying ocular disease include infectious endophthalmitis, sterile intraocular inflammation, rhegmatogenous retinal detachment, increased intraocular pressure and ocular hemorrhage. Drug-related dverse events which are influenced by underlying ocular disease include development or progression of tractional retinal detachment when IVB administration done before vitrectomy in patients with advanced proliferative diabetic retinopathy, possible increase in the incidence of retinal pigment epithelium tears in patients with AMD and worsening retinal detachment in patients with severe retinopathy of prematurity [9,12].

Preservative free vials of bevacizumab have not been created for intravitreal administration.A recent systematic review with aim of evaluating the safety of IVB monotherapy in adult ophthalmic conditions assessed 22 controlled trials and 67 observational studies that contained at least 10 patients showed no difference in the rate of endophthalmitis between the two groups of patients receiving off-labeled IVB or licensed IVR [13]. There are several methods for preparing IVB including multiple withdrawal from the same vial within the same day or at different days under aseptic conditions by ophthalmologist or preparing single use syringes of IVB injection from bevacizumab vials by a compounding pharmacy or a pharmaceutical company. Based on that review,visual loss was the most commonly reported ocular event in patients who received IVB. Nine observational studies reported visual loss with an incidence rate of 0 to 50%. The definition of visual loss was not clear in most of these observational studies. It seems that in these studies, visual loss occasionally was related to other adverse events such as intraocular or anterior chamber inflammation or retinal detachment. Therefore the authors of that review doubted whether visual loss happened due to IVB treatment or progression of the patients’ diseases. Infectious endophthalmitis was reported in 10 out of 13 observational studies with clinical incidence rate of 0-1%. In three out of 13 studies in which patients were treated with locally prepared IVB,the incidence rates of infectious endophthalmitis were 0.02%, 0.2%, and 0.8%. A higher rate of endophthalmitis (0.9%) was reported in the study in which IVB was prepared by a compounding pharmaceutical company [13]. That review concluded that IVB adverse effects are low and comparable with other intravitreal treatments, sham injection, and laser therapy [13].

A case-series from a private ophthalmology center from Hong Kong showed that using the same bevacizumab vial for maximum 10 consecutive injections with multiple aspiration of drug from the same vial under proper sterile technique and discarding remaining drug at the end of the day without overnight storage was safe. No cases of endophthalmitis were reported with IVB from that center [2]. A larger case series from Iran also showed no cluster of endophthalmitis following multiple withdrawal from the same vial within the same day with comparable risk of post-injection endophthalmitis with other intravitreal treatment modalities [14]. Recently, an Indian ophthalmology center reported a cluster of clinically-proven endophthalmitis in 6 out of 8 patients who received IVB by multiple pricks of the same bevacizumab vial (Avastin®, Genethech, Inc.) by different tuberculin syringes under aseptic precautions but not under the hood. The patients were treated with intravitreal antibiotic administration, but only three patients responded sufficiently. The culture from the bevacizumb vial that had been used for those patients and was kept refrigerated showed no microorganism growth after four days [15].

Artunay *et al.* reported endophthalmitis incidence rate of 0.066% after IVB, when multiple doses had been withdrawn from a single vial in an out-patient setting [16]. Other studies have also reported endophthalmitis possibly due to contamination during the compounding procedures of bevacizumab. Yamashiro *et al.* reported culture-negative endophthalmitis in 14 out of 19 consecutive cases after IVB from a single batch of bevacizumab [17]. Lee *et al.* reported two patients who received IVB on the same day that developed endophthalmitis with the same strains of *Serratia marcescens* suggesting contamination during drug compounding. However, they did not find any case of endophthalmitis in another group of patients who received IVB aspirated from the same vial that was reused for multiple consecutive injections just before each procedure and discarded on the same day [18]. Similarly, the Pan-American Collaborative Retina Study Group (PACORES) reported more frequent endophthalmitis in eyes injected using previously compounded aliquots than in eyes given injections from the same multidose vial that was reused appropriately (0.33 vs 0.04%) [19]. Unexpectedly,a higher frequency of endophthalmitis was reported in patients utilizing compounded aliquots by pharmaceutical company compared to re-utilization of a single vial under aseptic conditions [13]. Another cluster of infectious endophthalmitis with *Streptococcus mitis*/*oralis* has been reported in Maiami, Florida, United States in 12 patients. IVB syringes for these patients had been prepared by a compounding pharmacy in South Florida. The same microorganism was found in cultures prepared from unused syringes from this compounding pharmacy [20].

## Proposed strategies for IVB preparation

Although some studies have shown the stability and sterility of compounded bevacizumab at 4°C up to 15 days or even 6 months, however, microbial contamination still remains a major concern since bevacizumab vials are preservative free [21,22]. Multiple drawing from the same vial increases the risk of contamination possibly due to wiping rubber septum by fingers or a dirty gauze, rubber stopper leakage, poor aseptic technique (*e.g.* entering the vial without alcohol swabbing), injection of air into the vial before solution removal, using a contaminated needle or syringe for drug removal, and inappropriate storage durations and temperatures [23,24]. An extensive evaluation of IVB prepared by eleven US academic compounding pharmacies under good manufacturing practice facilities showed no microbial or endotoxin contamination. However, there were significant differences in bevacizumab concentrations between prepared aliquots even those prepared in the same compounding pharmacy. Bevacizumab concentrations were lower than those expected in prepared aliquots [25]. Some strategies have been reported to decrease the risk of IVB contamination. One proposed approach to minimize the risk of vial contamination compared to the multiple prick method is to insert a 25 gauge needle into the rubber cap of the vial by single prick and leaving the needle in place. The drug should be drawn into different 1 mL syringes by every time changing only the syringes not the needle. It is better to use this vial for one day without overnight storage. Each group of called patients should be small in number and for unilateral, not bilateral, IVB [2,15]. According to the United States Pharmacopeia (USP), the preparation of IVB aliquots from bevacizumab vials may be categorized to be in medium-risk level for sterile compounding. The quality assurance procedures that recommended to be considered include sterile preparation within a laminar-airflow workbench, routine disinfection, air quality testing to reach an International Organization for Standards (ISO) class 5 environment and annual evaluations of aseptic manipulation of all pharmacy staffs working in sterile compounding [2,26].

## Counterfeit Becacizumab

Medication counterfeiters are a major threat in drug market all over the world. Expensive drugs such as bevacizumab are at greater risk to be replaced with counterfeiters. Therefore vigilance is needed by physicians, drug distributors and governmental inspection agencies. Detections of counterfeit Avastin in theUnited States [27] and China [28] sparked worldwide concerns. Cheng and Wei have reported an outbreak of acute postoperative endophthalmitis in Shanghai, China after IVB. They mainly incriminated off-label IVB due to the potential risk of endophthalmitis. In their report, 55 out of 116 patients who received IVB from three bevacizumab vials showed endophthalmitis. However, upon testing that bevacizumab batches, Avastin manufacturer and an independent laboratory both showed that there was no bevacizumab in the vials. The cultures of ocular tissues of some of these patients also were negative for any bacteria or fungi. Visual acuities of all these patients were recovered by topical or systemic corticosteroid therapy [28]. Cheng’s and Wei’s concepts have been criticized by Sun *et al.* because each expensive drug, including approved IVR is at risk for counterfeiters [29]. Recently happening some adverse effects following IVB treatment in several patients in Iran prompted Ministry of Health and Medical Education of Iran to warn pharmacies of ophthalmology centers to prepare Bevacizumab vials from approved pharmaceutical ditribution companies to be safe from counterfeit bevacizumab (www.ifdona.ir. Lettre number 655/153229 of Food and Drug Organization of Ministry of Health and medical Education of Iran).

## Conclusion

There is growing body of articles on successful use of IVB for the treatment of proliferative retinopathies. Although the off-label use of a drug is not equivalent to off-evidence use of that drug, however, until providing single use, low content vials (1.25 or 2.5 mg) of bevacizumab concerns for aseptic conditions and good manufacturing process should be taken to account when preparing aliquots from available preservative free 100 mg/4 mL vials. It is more logical and cautious to perform unilateral not bilateral IVB injection for each patient at each procedur. Additionally, governmental inspection agencies have to be more precise for detection of Bevacizumab counterfeiters.
